# Bladder cancer trends and mortality in the brazilian public health system

**DOI:** 10.1590/S1677-5538.IBJU.2019.0198

**Published:** 2020-01-10

**Authors:** Frederico Timoteo, Fernando Korkes, Willy Baccaglini, Sidney Glina

**Affiliations:** 1 Faculdade de Medicina do ABC Santo AndréSP Brasil Disciplina de Urologia, Faculdade de Medicina do ABC, Santo André, SP, Brasil;; 2 Hospital Israelita Albert Einstein São PauloSP Brasil Divisão de Urologia, Hospital Israelita Albert Einstein, São Paulo, SP, Brasil

**Keywords:** Urinary Bladder Neoplasms, Mortality, Public Health

## Abstract

**Introduction:**

Considering the lack of data on BC trends in Brazilian population, mainly as a result of the difficulty on gathering data, the present manuscript provides an overview of bladder cancer incidence, hospitalization, mortality patterns and trends using the Brazilian Data Center for The Public Health System (DATASUS).

**Materials and Methods:**

All hospital admissions associated with BC diagnosis (ICD-10 C67) between 2008 and 2017 were analyzed. Distributions according to year, gender, age group, ethnicity, death, length of hospital stay, and costs were evaluated. Demographic data was obtained from the last Brazilian national census.

**Results:**

From 2008 to 2017 there were 119,058 public hospital admissions related to BC. Patients were mostly white males aged 60 to 79 years-old. Mortality rates for patients who have undergone surgery was 6.75% on average, being 7.38% for women and 6.49% for men. Mortality rates were higher when open surgeries were performed compared to endoscopic procedures (4.98% vs 1.18%). Considering only endoscopic procedures, mortality rates were three times higher after urgent surgeries compared to elective ones (2.6% vs 0.6%). Over the years the cystectomy/transurethral bladder resection (C/T) ratio significantly decreased in all Brazilian Regions. In 2008, the C/T ratio was 0.19, while in 2017 it reduced to 0.08.

**Conclusions:**

Despite BC relatively low incidence, it still represents a significant social economic burden in Brazil, as it presents with recurrent episodes that might require multiple hospitalizations and surgical treatment. The set of data collected might suggest that population access to health care has improved between 2008-2017.

## INTRODUCTION

According to American Cancer Society, in the United States in 2018, approximately 81, 190 patients were diagnosed and about 17.240 deaths occurred due to bladder neoplasm (1). Data retrieved from the Surveillance, Epidemiology and End Results Program from National Cancer Institute (SEER), recollects that rates for new bladder cancer (BC) cases have been falling on average 1.0% each year over the last 10 years. Nevertheless, BC remains as the 6^th^ more common type of cancer in the United States, accounting for 4.7% of all new cancer cases (2).

Worldwide, about 549.393 new diagnosis were made and 199.922 deaths were attributed to BC in 2018, which corresponds, respectively, to 3% of all cancer diagnosis and 2.1% of cancer-related deaths (3). In Brazil, where BC figures as the seventh most common malignancy in men and the 14^th^ in women, it is estimated a yearly diagnosis of 6.43 new cases per 100.000 men and 2.63 per 100.000 women (4). Whilst more frequent in men, BC tend to be found more clinically significant and in more advanced stages in women (5). As BC manifests primarily with gross or microscopic hematuria and has a recurrent pattern, patients who have been diagnosed with the disease frequently require multiple procedures and hospitalizations, both elective or urgent, in order to achieve either cure or at least control the disease. Despite having a relatively low incidence, clinical characteristics of this neoplasms results in a relevant social and economic burden to Brazilian government. In 2014 alone, 27.738 life years were lost due to BC deaths, US$ 29.879.165 were directly spent with BC treatment and an indirect loss of US$76.996.523 was estimated (6).

It is well known that the incidence of BC varies geographically with higher incidence rates in Western Europe and North America and lowest rates in Eastern Europe and Asia (7). There is data recollecting that the disease incidence may vary significantly even within a country, in the United States there are lower incidence rates in Utah and Hawaii compared to the Northeastern states (8). Studies assessing mortality and incidence of BC found that more than 60% of cases and over half of the deaths occur in less developed countries (3).

Bladder cancer occurrence is strongly related to environmental factors and aging. Smokers are 2-4 times more likely to develop BC and this association seems to be related to smoking intensity (9, 10).

Workplace exposure to carcinogens may account to approximately 10-20% of BC cases (11).

Metal workers, painters, rubber industry and textile workers and cement or mine workers are professionals with higher risk for BC due to exposure to polycyclic aromatic hydrocarbons (12, 13).

Considering social and demographic characteristics along with BC treatment impact on Brazilian Public Health System, it is imperative that caregivers be aware of BC epidemiology in the Brazilian population. The present manuscript provides an overview of BC incidence, hospitalization, mortality patterns and trends using the Brazilian Data Center for The Public Health System (DATASUS).

## MATERIALS AND METHODS

Brazilian Public Health System Information Database was used as the primary data source for our study. DATASUS represents the primary effort of Brazilian Federal Government to collect data from the national health system. This database includes information from all public health hospitals throughout the country, guaranteeing health support to about 170 million Brazilians.

All hospital admissions associated with BC diagnosis (ICD-10 C67) between 2008 and 2017 were analyzed. Distributions according to year, gender, age group, ethnicity, death, length of hospital stay and costs were evaluated.

Additionally, another search was performed according to surgical procedure. All procedures directly associated with BC surgery were evaluated, and divided into endoscopic surgery (codes: 0409010383-BLADDER TUMOR ENDOSCOPIC RESSECTION, 0416010172-ONCOLOGIC BLADDER TUMOR ENDOSCOPIC RESSECTION) and open surgery (codes: 0409010022-PARTIAL CYSTECTOMY, 0409010030-RADICAL CYSTECTOMY, 0409010049-SINGLE STAGE RADICAL CYSTECTOMY AND URINARY DIVERSION, 0409010057-CYSTOENTEROPLASTY, 0416010024-ONCOLOGIC SINGLE STAGE RADICAL CYSTECTOMY AND URINARY DIVERSION, 0416010032-ONCOLOGIC RADICAL CYSTECTOMY AND SIMPLE URINARY DIVERSION). For this second search, data evaluated included year, geographic region of Brazil, death rate, length of hospital stay and characteristic of hospital admission (elective vs. urgent).

Demographic data from the Brazilian population during the studied period were obtained from the last national census, realized in 2010 and trends in the rate of BC diagnosis and treatment from 2008 to 2017 were described.

Statistical analysis was performed using SPSS 13.0 (SPSS for Mac OS X, SPSS, Inc., Chicago, Illinois). Groups were compared with Pearson’s chi-square test and ANOVA. Statistical significance was determined at p <0.05.

## RESULTS

**Figure 1:**
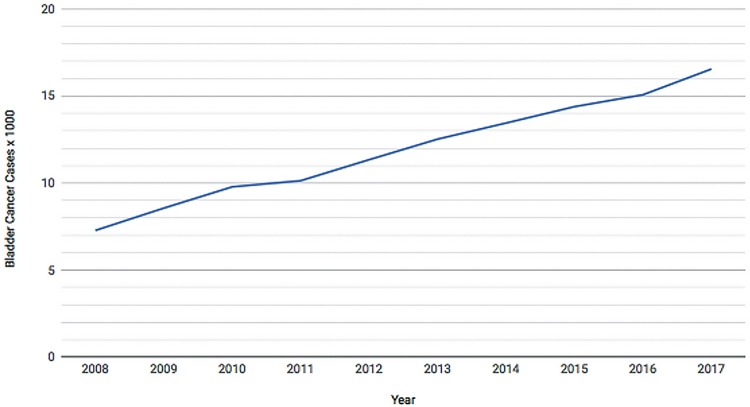
Bladder Cancer Admissions on Brazilian Public Health System (2008-2017).

**Figure 2:**
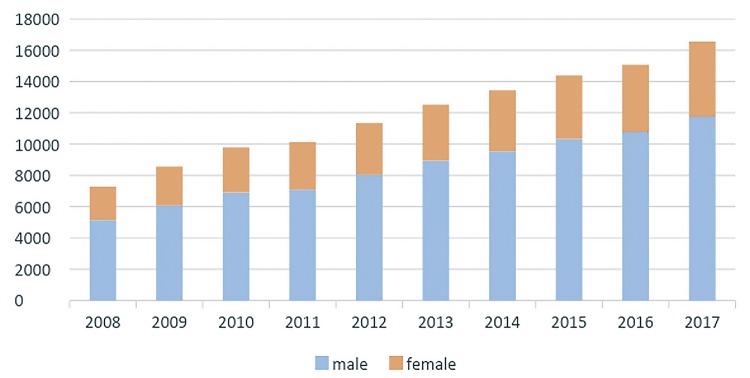
Bladder Cancer admissions according to gender (2008-2017).

**Figure 3:**
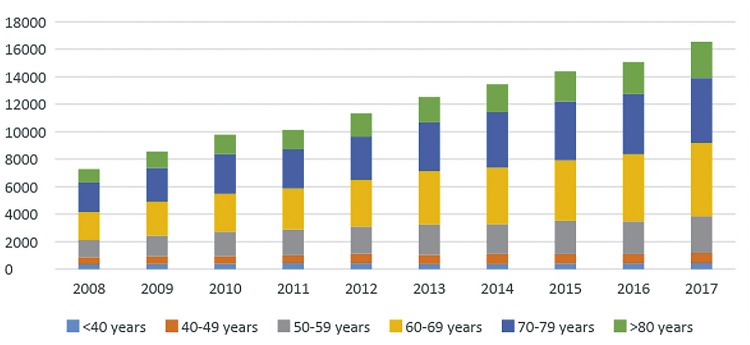
Bladder Cancer admissions according age group (2008-2017).

**Figure 4:**
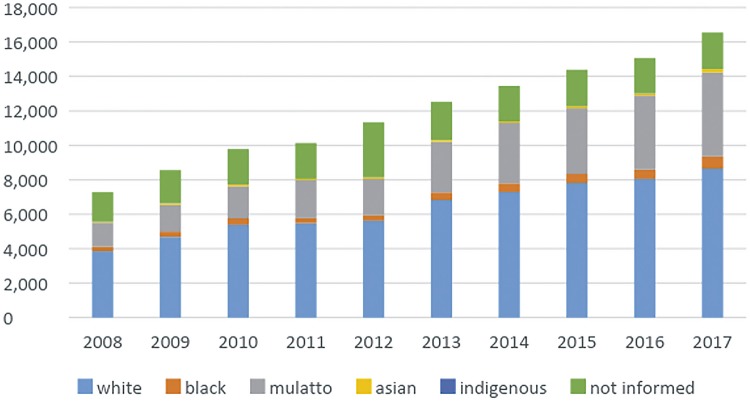
Bladder Cancer admissions according to ethnicity (2008-2017).

From 2008 to 2017, the population that relies on Brazilian Public Health System has increased 6.3% reaching 162 million people in 2017 and there were 119.058 patients admitted to public hospitals in Brazil with diagnosis of BC along those years. The number of hospital admissions related to BC went from 7.277 in 2008 to 16.547 in 2017. Patients admitted with BC were mostly white males aged 60 to 79 years. Figures 1-4 demonstrates admission growth tendency during the time of the study (Figure-1) and the distributions of these patients according to gender (Figure-2), age group (Figure-3) and ethnicity (Figure-4). The death rate among patients admitted with BC remained quite stable, being 6.4% in 2008 and 6.6% in 2017. Table-1 summarizes BC admissions, deaths and mortality rate.

**Table 1:** BC Admissions, Deaths and Mortality Rates from 2008-2017.

Year	Population using Public Health System	BC cases (n=119.058)	BC rate	BC deaths	Mortality rate
			(x10^-5^)		(%)
					
2017	162.077,403	16,547	10.21	1,100	6.6%
2016	160.022,071	15,069	9.42	1,032	6.8%
2015	156.750,009	14,387	9.18	1,005	7.0%
2014	154.265,099	13,445	8.72	879	6.5%
2013	153.223,204	12,521	8.17	873	7.0%
2012	152.901,741	11,340	7.42	762	6.7%
2011	152.746,237	10,133	6.63	651	6.4%
2010	151.858,919	9,781	6.44	666	6.8%
2009	153.659,783	8,558	5.57	615	7.2%
2008	152.427,207	7,277	4.77	466	6.4%

**Figure 5:**
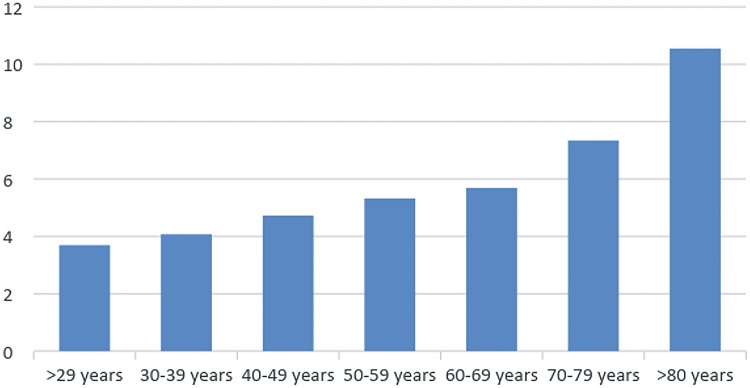
Bladder Cancer Mortality during hospitalization according to age group.

Across the studied years, among individuals who have undergone surgery, there was a 6.75% mortality rate, being 7.4% for women and 6.5% for men. Highest mortality rate occurred in 2008, when it reached 7.2% for all individuals and 7.7% for women along with 7.0% for men. BC mortality during hospitalization was higher among patients who were over 80 years-old (Figure-5).

During procedure analysis, it was found 77.353 endoscopic procedures to treat BC with 915 deaths and 8.925 open procedures with 441 deaths performed during the decade evaluated, which translates to a mortality rate of 1.2% and 5.0%, respectively, between 2008-2017 (Table-2). There were 54.759 elective transurethral bladder resections (TURb) during the studied period, with 326 documented deaths, while there were 589 deaths among the 22.595 urgent TURb, corresponding respectively, to a mortality rate of 0.6% and 2.6% (Table-3). Mortality rates for urgent radical cystectomies were about 50% higher than the rate for elective radical cystectomies (9.74 vs. 6.02).

**Table 2:** Endoscopic vs Open Procedures and related Death rates.

	TURB			Radical Cystectomies		

Year	Cases	Deaths	Rate	Cases	Deaths	Rate
2008	5,495	72	1.31%	1,053	38	3.61%
2009	5,814	67	1.15%	1,099	52	4.73%
2010	6,313	62	0.98%	982	41	4.18%
2011	6,632	62	0.93%	875	42	4.80%
2012	7,053	96	1.36%	795	43	5.41%
2013	8,205	109	1.33%	883	47	5.32%
2014	8,836	117	1.32%	858	54	6.29%
2015	9,028	120	1.33%	800	37	4.63%
2016	9,496	99	1.04%	730	33	4.52%
2017	10,481	111	1.06%	850	54	6.35%

**Total**	**77,353**	**915**	**1.18%**	**8,925**	**441**	**4.98%**

**Table 3:** Deaths after TURb, elective vs. urgent.

Deaths after TURb (elective vs urgent)						

	Elective			Urgent		
	
	Surgeries	Deaths	Rate (%)	Surgeries	Deaths	Rate (%)
2008	3,627	29	0.8	1,868	43	2.3
2009	3,950	27	0.684	1,864	40	2.15
2010	4,416	22	0.498	1,897	40	2.1
2011	4,720	19	0.403	1,912	43	2.24
2012	5,026	29	0.577	2,027	67	3.3
2013	5,954	47	0.789	2,251	62	2.8
2014	6,309	43	0.682	2,527	74	2.9
2015	6,382	37	0.58	2,646	83	3.1
2016	6,850	32	0.467	2,646	67	2.5
2017	7,525	41	0.545	2,956	70	2.36

**Total**	**54,759**	**326**	**0.595**	**22,594**	**589**	**2.6**

The open procedures were mostly radical cystectomies, being 1.966 (22.0%) partial cystectomies. About 4.209 (47.2%) of the open procedures were performed in Brazilian Southeast Region, followed by South, Northeast, Midwest and North regions with 1.935 (21.7%), 1.403 (15.7%), 742 (8.3%) and 636 (7.1%) of these surgeries, respectively.

Mean length of hospital stay after was 6.9 days for partial cystectomies and 13.6 days for radical cystectomies. Patients who have undergone surgeries in North Region presented the lowest mean hospital stay of 10.3 days followed by patients from South (11.11), Northeast (12.07), Midwest (12.82) and Southeast (13.27) regions, respectively. For TURb, mean length of hospital stay was of 4.3 days.

**Figure 6:**
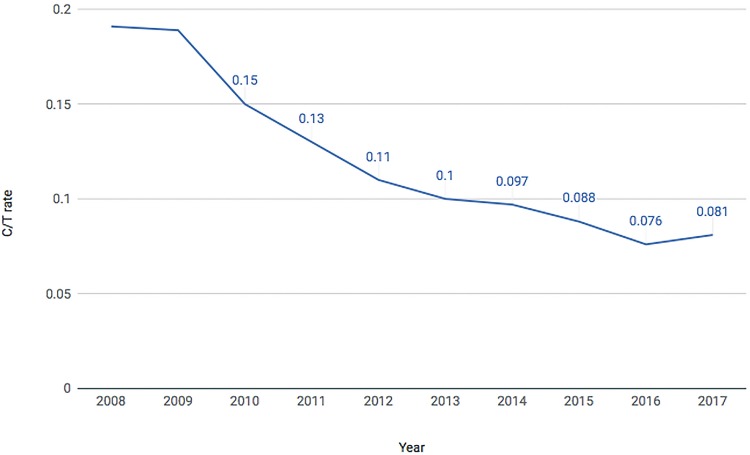
Cystectomy over TURb Rate from 2008 – 2017.

During the decade evaluated, the correlation between radical cystectomy and TURb (C/T ratio) presented a significant reduction. In 2008, the C/T ratio was 0.19, while in 2012 it reduced to 0.11 and then to 0.08 in 2017 (Figure-6).

## DISCUSSION

Recent studies have reported that worldwide BC incidence trends towards stabilization or decline in men versus increase in women with an overall decrease in mortality. In Brazil there is paucity of data, but Antonini et al. demonstrated similar trends regarding incidence whilst there was a slightly increase in mortality (14). Our study has some important findings.

First, during the studied decade the number of hospital admissions related to BC has significantly increased. There were 7.277 admissions in 2008 and in 2017 16.547 were recorded. Even though this finding could be attributed to an increase in BC incidence, it is our belief that better access to health systems and better notification also explains this increment. There are some findings that reinforce the latter hypothesis. Although there was a large number of admissions, death rates among patients admitted with BC related diagnosis remained stable: 6.4% in 2008 vs. 6.6% in 2017. Additionally, the number of BC admissions more than doubled while the number of individuals that rely on Brazilian Public Health System has increased only 6.3%.

**Figure 7:**
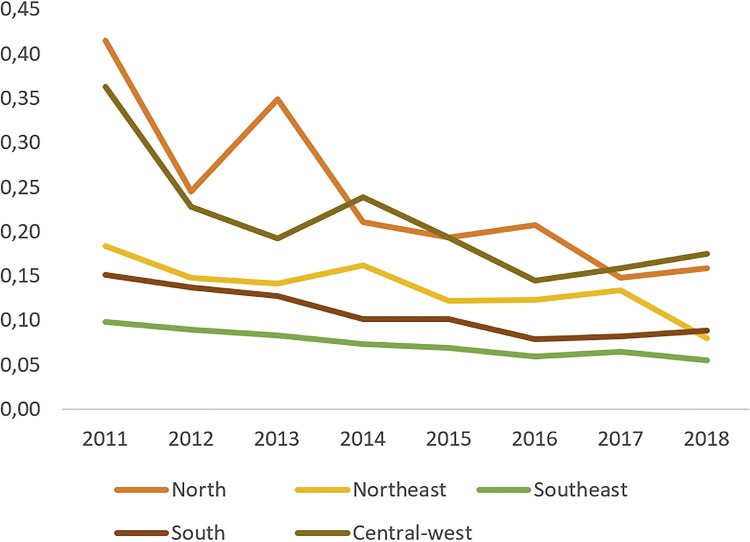
Cystectomy over TURb Rate from 2008 – 2017 according to geographic regions in Brazil.

Second, it is important to highlight our findings regarding the C/T ratio for BC treatment. Across the years the C/T ratio has decreased from 0.19 in 2008 to 0.08 in 2017. It is our understanding that in an ideal scenario the vast majority of bladder tumors would be treated in early stages by endoscopic procedures. Thus, the C/T ratio decrease might point that early diagnosis might have been performed and more superficial BC cases were treated vis a vis invasive disease. Access to Public Health System might have improved since not only the number of individuals admitted to manage BC has significantly increased but also the proportion of cystectomies has decreased, which, as mentioned before, may reflect an improvement of BC treatment in early stages with TURb. The C/T ratio might also reflect regional disparities in Brazil. Over 68% of the cystectomies were performed in Southeast and South regions; those Brazilian regions present the highest Human Develop Index (HDI) (15). In these southern regions, C/T ratio was lower than that presented in underdeveloped northern regions of the country (Figure-7). This finding might be related to better access to health care along with more equipped hospitals in these regions leading to higher diagnosis rates and adequate treatment. We acknowledge, however, that definite conclusions should not be made regarding the C/T ration as this is not a validated index and further studies encompassing different populations and in a more controlled scenario are needed to evaluate the usability of the C/T ratio as a quality of care index. Figure-7 demonstrates the C/T ratio in the five geographic regions of Brazil during the last years.

Third, there were some factors associated with increased mortality following BC treatment. Mortality significantly increased with age (Figure-5), which, according to literature, may reflect late BC stages at diagnosis and poorer biologic response for both the disease and the morbidity of its treatment (16). This increment in mortality in the elderly population might become progressively more relevant, since we have also observed that population aging process can be translated into a progressively higher number of octogenarians being diagnosed and treated for BC. A large SEER database analysis of over 10.000 patients treated in the US with RC between 1984 and 2004 showed that at 90 days mortality is 1% for patients younger than 60 years of age, 6% between ages 69 and 83 and 14% for patients over age 89 (17).

Cumberbatch et al., at a recent systematic review, reported that mortality varies worldwide but trends to be higher in men than in women (18). According to SEER database, despite 30-day mortality after radical cystectomy was higher for men, 90-day mortality became higher for women (4.8% Vs. 3.2%) (17). In accordance, our data, shows higher BC mortality among women vs. men (7.38% Vs. 6.49%).

In addition, surgeries performed as an urgent basis burdened a significant higher mortality rate than elective procedures (2.1% vs. 0.4%, p <0.0001). Likewise, higher mortality relative rates occurred following radical cystectomies performed urgently vs. elective (9.74 vs. 6.02, p <0.0001). The reported rate of perioperative mortality after radical cystectomy ranges from 0.8% to 5.6% in foreign contemporary cystectomy series (17). Mortality rates in the current Brazilian series was somewhat higher than that. Several variables may affect this rate, including patient age, nutrition status, tumor characteristics/advanced stages at diagnosis and the lack of large volume centers. This elevated mortality rates after BC treatment in Brazil is still to be studied in detail as several factors might have influence on this finding: from the data acquisition method to problems related to health care access, especially for the poorer living in remote areas of Brazil.

Fourth, partial cystectomies rate was high, representing 22% of all open procedures. It has been previously demonstrated in the SEER database that while high volume centers perform partial cystectomies in 12% of patients, low volume centers tend to perform partial cystectomies in as much as 34% of all open procedures. These high rates might suggest that many patients are being inadequately treated for their diseases (19).

Our study has some limitations. As an epidemiologic study, definitive conclusions cannot be made based exclusively on our findings. Like other neoplasms, BC might still be underreported as cause of hospitalization and death, especially in developing countries as Brazil. Additionally, admissions are not considered in an individual basis, and patients can be considered twice in data analysis. The C/T ratio is also not a validated tool and might be biased by several distinctive factors. However, mainly as a result of the difficulty to gather these data, there is a paucity of data of epidemiologic studies in BC in Brazil. Therefore, our study outlines BC trends in Brazilian population in the last decade and might bring insights to help establish public health policies.

## CONCLUSIONS

In Brazil, BC incidence seems to corroborate the current literature when it comes to gender and ethnicity. Furthermore, despite BC relatively low incidence, it still represents a significant social economic burden in Brazil, as it presents with recurrent episodes that might require multiple hospitalizations and surgical treatment. Present data might suggest that population access to health care and notification have improved between 2008-2017, with significant growth in the number of hospital admissions related to BC. Considering the impact of BC in Brazilian Public Health system, our data reinforces the importance of further studies and policies on BC risk factors and treatment.
